# Vitamin B12 Supplementation Improves Oocyte Development by Modulating Mitochondria and Yolk Protein in a Caffeine-Ingested *Caenorhabditis elegans* Model

**DOI:** 10.3390/antiox13010053

**Published:** 2023-12-28

**Authors:** Hyemin Min, Mijin Lee, Sangwon Kang, Yhong-Hee Shim

**Affiliations:** Department of Bioscience and Biotechnology, Konkuk University, Seoul 05029, Republic of Korea; mintmin@konkuk.ac.kr (H.M.); miranda12@konkuk.ac.kr (M.L.); cjstlakstl@konkuk.ac.kr (S.K.)

**Keywords:** caffeine, vitamin B12, yolk protein, mitochondria, oogenesis, *Caenorhabditis elegans*

## Abstract

Vitamin B12 is an essential cofactor involved in the function of two enzymes: cytosolic methionine synthase and mitochondrial methylmalonic-CoA mutase. In our previous studies, caffeine (1,3,7-trimethylxanthine), the most popular bioactivator, was shown to reduce yolk protein (vitellogenin) and fertility in a *Caenorhabditis elegans* model. Based on the previous finding that methionine supplementation increases vitellogenesis in *C. elegans*, we investigated the role of vitamin B12 in methionine-mediated vitellogenesis during oogenesis in caffeine-ingested animals (CIA). Vitamin B12 supplementation improved vitellogenesis and reduced oxidative stress by decreasing mitochondrial function in CIA. Furthermore, the decreased number of developing oocytes and high levels of reactive oxygen species in oocytes from CIA were recovered with vitamin B12 supplementation through a reduction in mitochondrial stress, which increased vitellogenesis. Taken together, vitamin B12 supplementation can reverse the negative effects of caffeine intake by enhancing methionine-mediated vitellogenesis and oocyte development by reducing mitochondrial stress.

## 1. Introduction

Caffeine (1,3,7-trimethylxanthine) belongs to the methylxanthine group and is the most popular compound consumed worldwide in the form of tea, coffee, and caffeinated soft drinks. Studies on caffeine intake in animal models have shown that both beneficial and harmful effects on animal physiology depend on age and dose [1,2,3]. The complex effects of caffeine make it difficult to understand its mode of action. To circumvent this problem, a simple animal model in which the physiological effects of caffeine can be analyzed is necessary. For this purpose, *Caenorhabditis elegans* is an excellent animal model because it shows an apparent phenotype and has a simple organ arrangement, and in vivo analysis at the organismal level is manageable [4]. A high dose of caffeine (>10 mM) mainly causes adverse effects [5,6,7], whereas a low dose of caffeine (<10 mM) generally has beneficial effects [8]. We previously found that caffeine intake at the young adult stage of *C. elegans* at a low dose (<10 mM) reduced fertility, with defective oocytes resulting from decreased levels of yolk protein [9]. However, the molecular mechanisms underlying the effects of caffeine on oocyte development remain unclear.

*C. elegans* oogenesis provides an ideal system for characterizing a link between the metabolic state and oocyte development. During *C. elegans* oogenesis, oocyte precursor germ cells, which are located proximal to the distal proliferative region of the two gonad arms, enter meiosis I and II and produce an oocyte with one copy of each chromosome [10]. Simultaneously, a large amount of cytoplasm accumulates in the proximal region of the gonad, which mainly contains yolk proteins for early embryonic development [11]. We previously showed that maternal caffeine intake reduced vitellogenesis and reproduction, and increased embryonic lethality in a *C. elegans* model [9]. The strong relationship between metabolism and oocyte maturation is mediated by mitochondrial activity in *Drosophila* and *Xenopus* [12]. Moreover, female metabolic diseases cause infertility and polycystic ovary syndrome in humans, suggesting an important link between metabolic regulation and oocyte development [13,14]. Despite its significance, the effects of a habitual diet on normal oocyte development and mitochondrial function remain largely unknown.

**Figure 1:**
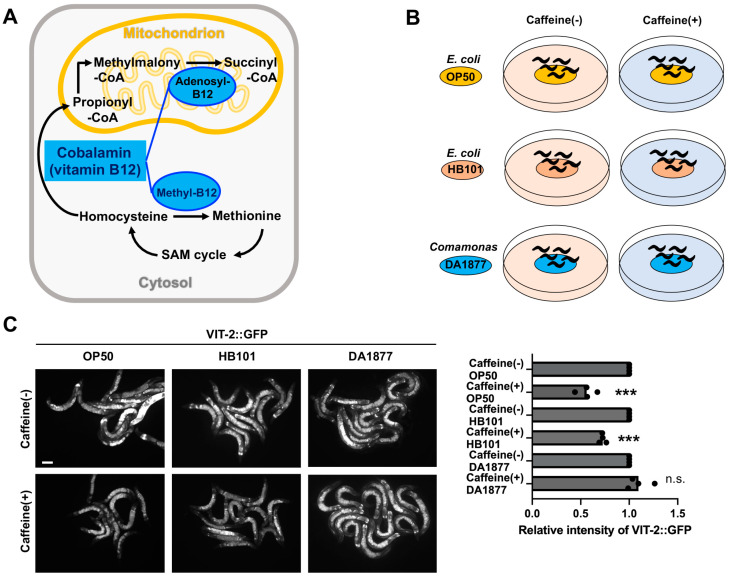

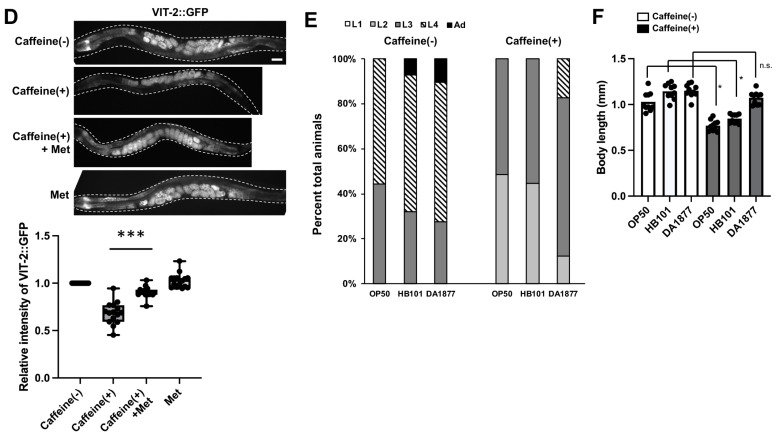
Effects of a bacterial vitamin B12 diet on vitellogenin (yolk protein) and larval development in caffeine-ingested animals (CIA). (**A**) Vitamin B12-dependent pathways in the mitochondrion and cytosol. (**B**) An experimental scheme for the effect of three different bacterial diets on CIA. (**C**) Representative images and graphs show the intensity of VIT-2::GFP in OP50, HB101, and DA1877 bacteria under caffeine or caffeine-free conditions. Bar, 50 μm. (**D**) Representative images and graphs show the intensity of VIT-2::GFP with or without methionine (Met) treatment in CIA. Bar, 25 μm. (**E**,**F**) Developmental growth (**E**) and body length (**F**) in animals fed with or without caffeine under OP50, HB101, and DA1877 bacteria. Data represent mean ± standard deviation. n.s., *p* > 0.5. *, *p* < 0.05. ***, *p* < 0.001. Number of analyzed animals, *n* ≥ 25 in respective conditions.

Mitochondria have essential metabolic functions and are major sites of energy production in animals. The maintenance of mitochondrial homeostasis and integrity is critical for cellular function and survival. In addition to energy production, mitochondria are involved in cellular metabolism and signal transduction [15]. Mitochondrial biogenesis, function, and antioxidant responses are closely associated with dietary conditions, including amino acid restriction [16]. Methionine metabolism has a strong impact on oxidative stress and mitochondrial function [17]. In addition, methionine improves egg production in chickens [18] and milk production in dairy cows [19], suggesting that it plays an important role in nutrient metabolism in animal reproduction. Interestingly, methionine supplementation increases vitellogenesis to mitigate the negative effects of calorie restriction in *C. elegans* [20]. In methionine metabolism, vitamin B12 (cobalamin) serves as a methyl group carrier in the methyltetrahydrofolate-homocysteine methyltransferase enzyme, also known as methionine synthase, and as a cofactor for mitochondrial methylmalonyl-CoA mutase (Figure 1A) [21,22]. The essential role of vitamin B12 in methionine production is emerging because methionine is further converted to S-adenosylmethionine, which is the most important cellular methyl donor [22]. Therefore, disruption of methionine metabolism has implications for all methylation-dependent cellular reactions, including cell proliferation, differentiation, and epigenetic modifications [22,23,24].

In this study, we aimed to elucidate the beneficial role of vitamin B12 in mitigating the negative effects of habitual caffeine intake on oocyte development, vitellogenesis, and mitochondrial function.

## 2. Materials and Methods

### 2.1. C. elegans Strains and Maintenance

*C. elegans* strains were maintained at 20 °C on nematode growth medium (NGM) agar plates as previously described [25]. To examine the effects of caffeine intake, 10 mM caffeine (Sigma-Aldrich, St. Louis, MO, USA) was added to NGM before autoclaving, as previously described [9]. Synchronized L4-stage animals were exposed to caffeine for 24 h at 20 °C, and adult-stage (24 h post-L4 stage) animals were examined. Then, 0.64 nM of vitamin B12 (Sigma-Aldrich, St. Louis, MO, USA) was added to the NGM plates. To examine the effect of a high-peptone diet on caffeine treatment, 500 mg/mL peptone (VWR International, Radnor, PA, USA) was added before autoclaving the basal medium. N2 (Bristol) was used as the wild-type strain. Animals were fed a diet of *Escherichia coli* OP50 unless otherwise noted. The following transgenic animals were used in this study: DH1033: *bIs1* [*vit-2*::GFP + *rol-6(su10060)*] X to observe vitellogenin gene 2 (*vit-2*) (NM060200) expression [26], SJ4100: *zcIs13* [*hsp-6*::GFP] to observe a mitochondrial stress response using heat shock protein 6 (*hsp-6*) (NM001383301.1) expression [27], and EGD623: *egxSi152* [*mex-5p: tomm-20::gfp::pie-1 3′UTR + unc-119(+)*] II; *unc-119(ed3)* III to observe mitochondrial morphology using *tomm-20* (NM073691) expression [28].

### 2.2. Bacterial Strains

*E. coli* OP50, *E. coli* HB101, and *Comamonas aq*. DA1877 were obtained from the *Caenorhabditis elegans* Genetics Center (University of Minnesota, 6-160 Jackson Hall, 321 Church street S.E., Minneapolis, MN, USA). For the bacterial experiments, *Comamonas aq*. DA1877 was cultured overnight in LB Broth medium at 37 °C, and then incubated at 80 °C for 120 min.

### 2.3. N-Acetyl-L-cysteine (NAC) Treatment

NAC (Sigma-Aldrich, St. Louis, MO, USA) was used to examine the effect of antioxidants on the effects of caffeine treatment. Synchronized L4-staged animals were placed on NGM plates containing 5 mM NAC with either 0 mM or 10 mM caffeine NGM for 24 h at 20 °C, and then used for further experimentation.

### 2.4. Live Imaging of Fluorescently Labeled Animals

For live imaging under a fluorescence microscope (Zeiss Axioscope, Oberkochen, Germany), 10–20 transgenic animals were placed in 10 µL of M9 buffer containing 0.2 mM levamisole on a poly L-lysine-coated glass slide and covered with a coverslip. Raw images were analyzed using Fiji/ImageJ software (v1.53).

### 2.5. Analysis of Developmental Rate and Body Length

We measured the developmental rate of a population by synchronizing animals with L1-stage arrest at M9 for 24 h and then allowing animals to develop for 48 h at 20 °C under each dietary condition. The developmental rate was measured by the percentage of larvae of the total number of hatched embryos that reached each developmental stage, as previously described [5]. To examine body length, the synchronized animals were mounted on agar pads in 0.2 mM levamisole, imaged under Nomarski optics, and photographed. Images were analyzed using Fiji/ImageJ (v1.53) to determine length measurements.

### 2.6. DNA Staining

DNA staining was performed to observe oocyte development in caffeine-ingested animals (CIA). Briefly, the animals were fixed with 4% paraformaldehyde at 20 °C for 20 min and further fixed with 70% ethanol at 4 °C overnight. The specimens were stained with DAPI (Thermo Scientific, Karlsruhe, Germany) to stain the DNA and observed under a fluorescence microscope (Zeiss Axioscope, Oberkochen, Germany).

### 2.7. Analysis of Reactive Oxygen Species (ROS) Production in Mitochondria

To examine the effect of caffeine intake on mitochondrial ROS in oocytes, CellROX Green (Invitrogen, Carlsbad, CA, USA) staining was performed as described previously [29]. Briefly, the CellROX Green solution (5 mM stock) was prepared and diluted at a 1:500 in M9. The animals were transferred to each condition containing the staining solution and stained at 20 °C. The animals were mounted on poly-L-lysine-coated glass slides and imaged using a fluorescence microscope (Zeiss Axioscope, Oberkochen, Germany). Relative quantification of mitochondrial ROS was analyzed by Fiji/ImageJ software (v1.53).

### 2.8. Analysis of Mitochondrial Membrane Potential (MMP)

To measure mitochondrial membrane potential, tetramethylrhodamine methyl ester (TMRM; Thermo Fisher Scientific, Waltham, MA, USA) staining was performed, as previously described [29]. After staining, the animals were mounted on poly-L-lysine-coated glass slides and imaged using a fluorescence microscope (Zeiss Axioscope, Oberkochen, Germany). Relative fluorescence intensity was analyzed by Fiji/ImageJ (v1.53).

### 2.9. RNA Interference (RNAi)

Two RNAi methods were used as previously described [30,31]. The dsRNA for the *vit-2* gene was synthesized in vitro using cDNA templates. cDNA templates flanked by T7 promoter sequences were generated by PCR using the T7 primer (5′-GTAATACGACTCACTATAGGGC-3′) and the CMo422 primer (5′-GCGTAATACGACTCACTATAGGGAACAAAAGCTGGAGCT-3′). A soaking buffer without dsRNA was used as a negative mock RNAi control. The L4-stage animals were soaked in dsRNA solution for 24 h, transferred to plates to be grown for 24 h, and the adult-stage animals were examined. Depletion of *cco-1* was performed using the bacteria-mediated (feeding) method with *E. coli* HT115(DE3) containing the F26E4.9 (*cco-1*)-inserted L4440 vector. The L4440 empty vector in HT115 cells was used as an RNAi control.

### 2.10. Statistical Analysis

Statistical analyses were performed using GraphPad Prism 9.3.1 software. *p* values lower than 0.05 were considered statistically significant. Data graphs were plotted in GraphPad Prism using scatter dot plots with bars or violin plots displaying the standard deviation from the mean value. *p* values are shown as asterisks, and *n* values for each experiment are denoted in the corresponding figure legends.

## 3. Results

### 3.1. A Bacterial Vitamin B12 Diet Improves Vitellogenesis and Developmental Growth in Caffeine-Ingested C. elegans

Vitellogenesis (yolk protein production) is essential for reproduction during animal development, and there is evidence that this process is well controlled in terms of where, when, and how much protein is produced in *C. elegans*. We have previously shown that caffeine intake significantly reduces vitellogenesis [9]. Recently, methionine has been suggested to play a key function during vitellogenesis in dietary restriction [20]. Therefore, we investigated whether methionine metabolism and vitellogenesis were regulated by caffeine intake in *C. elegans*. The bacterial diet was changed from OP50 to DA1877. DA1877 is a vitamin B12-producing bacterial strain (*Comamonas* DA1877) in contrast to OP50, which is an *E. coli* strain commonly used as a food source for *C. elegans* in laboratory settings (Figure 1B). We also included HB101, another commonly used *E. coli* strain, which has altered levels of carbohydrate and fatty acid composition compared to OP50 as a control group with different micronutrients [32]. Vitamin B12 is an essential cofactor that functions in the methionine/S-adenosylmethionine (Met/SAM) cycle, which is a part of the one-carbon metabolism in the cytosol and the propionate catabolic pathway in the mitochondria (Figure 1A). We hypothesized that the reduced yolk protein levels in CIA could be recovered by administering DA1877. We observed vitellogenin gene 2 (*vit-2*) expression (VIT-2::GFP) in CIA fed with different bacterial diets, including *E. coli* OP50, HB101, and *Comamonas* DA1877, using the transgene *vit-2*::GFP, and we found that animals fed *Comamonas* DA1877 showed increased levels of VIT-2::GFP expression compared to the animals fed with *E.coli* OP50 or HB101 (Figure 1C). These results suggest that vitamin B12 supplementation improves vitellogenesis in CIA.

Next, we examined whether methionine supplementation could improve caffeine-induced reduction in vitellogenesis in *C. elegans*. Adult-stage animals expressing the transgene *vit-2*::GFP were observed after caffeine treatment, with or without methionine supplementation. VIT-2::GFP expression was increased by methionine supplementation in the CIA (Figure 1D), suggesting that a vitamin B12 diet improves methionine-mediated vitellogenesis in CIA.

We previously found that caffeine intake affects the development and growth of *C. elegans*. Therefore, we further examined the possible changes in developmental rate and growth by feeding CIA with DA1877, a vitamin B12-producing bacteria. We measured the developmental rate and body length of CIA-fed different bacterial diets. Surprisingly, animals grown in DA1877 with caffeine treatment showed an enhanced developmental rate compared with animals grown in either OP50 or HB101 with caffeine treatment (Figure 1E). We also observed that animals grown in DA1877 with caffeine treatment showed significant recovery in body length (Figure 1F). Based on these findings, a vitamin B12 diet appears to improve vitellogenesis and developmental growth by modulating methionine metabolism in CIA.

### 3.2. Vitamin B12 Supplementation Recovers Vitellogenesis and Mitochondrial Stress Response in Caffeine-Ingested C. elegans

**Figure 2:**
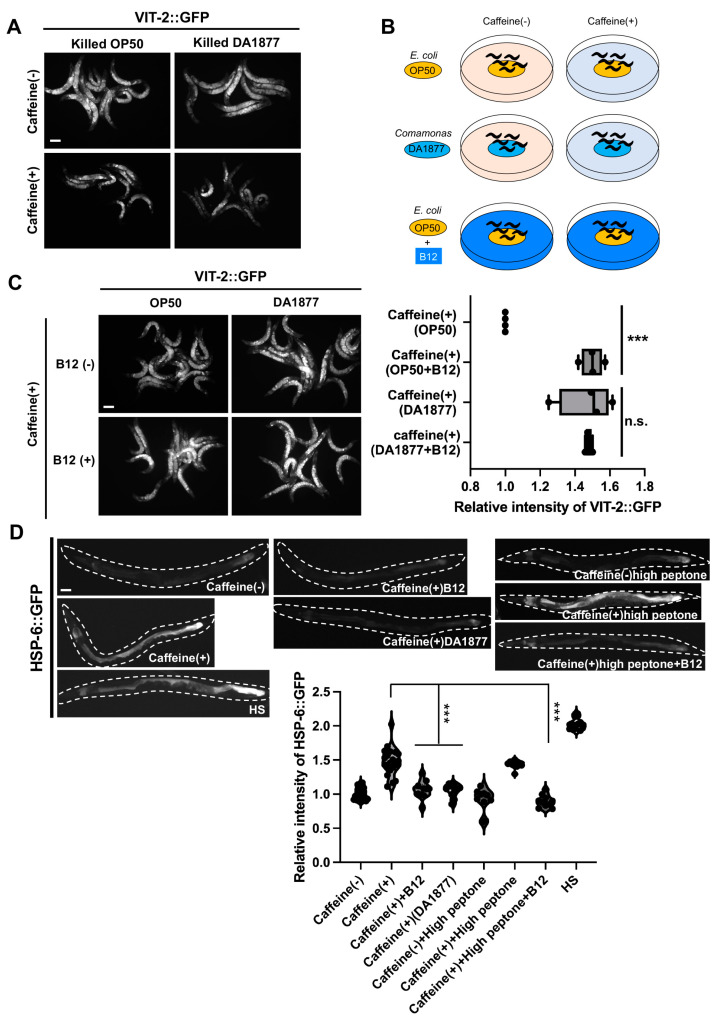
Effects of vitamin B12 supplementation on vitellogenin and mitochondrial stress response in CIA. (**A**) Representative images show that VIT-2::GFP transgenic animals were synchronized at the L4 stage and fed with killed OP50 and DA1877 bacteria with or without caffeine for 24 h at 20 °C. Bar, 50 μm. (**B**) An experimental scheme for effects of vitamin B12 on CIA. (**C**) Comparison of the intensity of VIT-2::GFP in OP50- or DA1877-fed CIA with or without B12 supplementation. Bar, 50 μm. (**D**) Comparison of the intensity of HSP-6::GFP in CIA fed with OP50 or DA1877 with or without vitamin B12, or with high peptone. Heat stress (HS) was used as a positive control. Bar, 25 μm. Data represent mean ± standard deviation. n.s., *p* > 0.5. ***, *p* < 0.001. Number of analyzed animals, *n* ≥ 25 in respective conditions.

To test whether vitamin B12 production by live bacteria recovered the reduced vitellogenesis caused by caffeine intake, we examined the effects of caffeine intake in killed DA1877 bacteria, and we found that VIT-2::GFP expression was similar to that in the OP50 diet with caffeine treatment (Figure 2A). This finding confirmed that a vitamin B12 supply is necessary to recover vitellogenesis in CIA. We next tested whether vitamin B12 supplementation in a standard OP50 diet had a similar effect (Figure 2B). In the OP50-fed CIA with vitamin B12, the expression of VIT-2::GFP was significantly higher than that in the control animals (Figure 2C). Moreover, no additional increase in the expression of VIT-2::GFP was observed in animals with vitamin B12 supplementation in the DA1877 diet with caffeine treatment (Figure 2C). These results indicate that the vitamin B12 supply in the *Comamonas* DA1877 diet is sufficient to improve vitellogenesis in CIA.

Vitamin B12 is required for mitochondrial function and is an essential cofactor in the conversion of methylmalonyl-CoA into succinyl-CoA. In our previous study, caffeine intake caused alterations in mitochondrial activity and morphology in intestinal cells with an increase in the expression of the mitochondrial stress response gene *hsp-6* in *C. elegans*. Therefore, we examined the expression of *hsp-6*::GFP, a mitochondrial stress response reporter gene, to determine whether vitamin B12 supplementation or a vitamin B12 bacterial diet can improve caffeine-induced mitochondrial alterations. Supplementation with vitamin B12 or DA1877 repressed HSP-6::GFP expression in CIA compared to that in the OP50-fed CIA (Figure 2D). These results suggest that vitamin B12 plays a role in mitochondrial stress resistance. Considering that vitamin B12 plays a crucial role in the maintenance of one-carbon metabolism with folate and methionine, and thus provides a better nutrient environment, we tested the effects of a high-peptone diet to determine whether improved nutrient value can confer resistance to stress induced by caffeine intake. Interestingly, when a high-peptone diet was supplied to CIA, a mitochondrial stress response was still observed (Figure 2D). However, animals fed the high-peptone diet supplemented with vitamin B12 showed a significantly reduced mitochondrial stress response (Figure 2D). Taken together, these results suggest that the caffeine-induced mitochondrial stress is effectively ameliorated by vitamin B12 and that caffeine-induced effects on vitellogenesis and mitochondria act through vitamin B12-dependent metabolic pathways.

### 3.3. Vitamin B12 Lessens Defective Oocyte Development Caused by Alterations in Mitochondrial Morphology and Function in Caffeine-Ingested C. elegans

**Figure 3:**
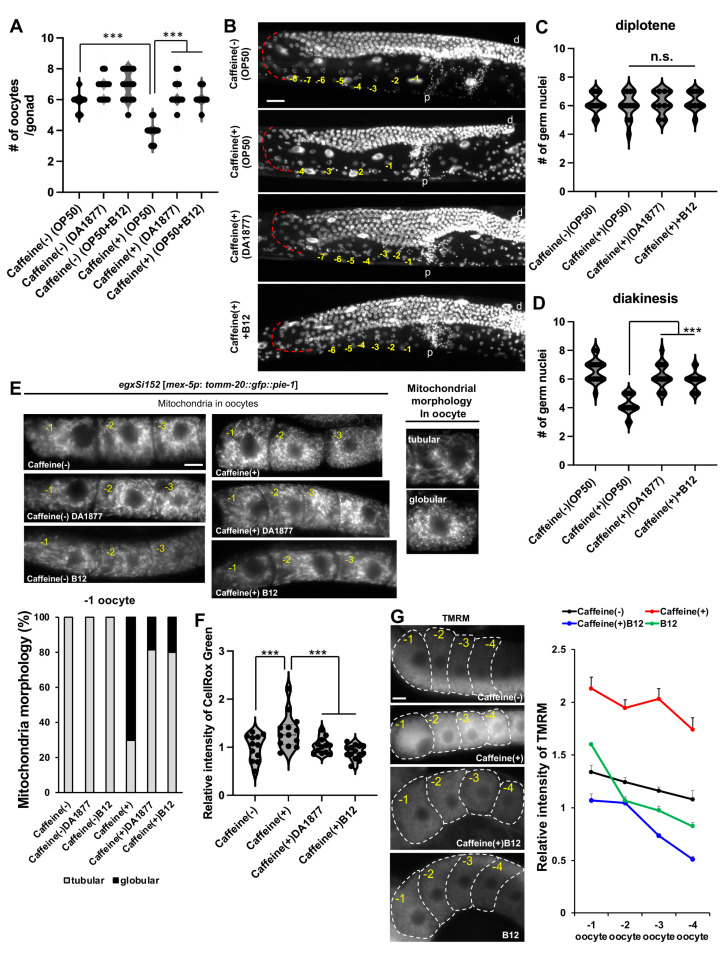
Effects of vitamin B12 on oogenesis in CIA. (**A**) Quantification of the number of oocytes produced by CIA fed with OP50 or DA1877 with or without vitamin B12. (**B**–**D**) Representative gonad images of DAPI staining (**B**) show the number of germ nuclei in diplotene (**C**) and diakinesis (**D**) in CIA fed the corresponding bacterial diet with vitamin B12 supplementation. The red dotted line indicates diplotene. Bar, 20 μm. The yellow-colored number indicates the developing oocytes that aligned from the proximal region; d, distal side of the gonad arm; p, proximal side of the gonad arm. (**E**) Comparison of mitochondrial morphology in the oocytes of caffeine-ingested EGD623 (*egxSi152 [mex-5*p*: tomm-20*::*gfp*::*pie-1]*) transgenic animals fed OP50 or DA1877 and supplemented with or without vitamin B12. The type of mitochondrial morphology was classified as tubular or globular. Bar, 5 μm. (**F**,**G**) Comparison of mitochondrial reactive oxygen species through CellROX Green staining (**F**) and mitochondrial membrane potential through TMRM staining (**G**) in the oocytes of CIA fed with OP50 or DA1877 supplemented with or without vitamin B12. Bar, 5 μm. The violin plot shows the relative level of intensity of CellRox Green (**F**). The line graph shows the relative intensity of TMRM in oocytes. Data represent mean ± standard deviation. n.s., *p* > 0.05. ***, *p* < 0.001. Number of analyzed animals, *n* ≥ 20 in respective conditions.

Yolk proteins are a major component of oocytes, and mitochondria are key organelles that control oocyte development in *C. elegans*. Thus, we measured the number of oocytes produced by *C. elegans* grown in the OP50, DA1877, OP50 + B12, or DA1877 + B12 diets with or without caffeine treatment. The OP50-fed CIA showed a significant reduction in the number of oocytes compared to animals fed a caffeine-free diet (Figure 3A). However, CIA fed either the DA1877 diet or the diet supplemented with vitamin B12 showed a significant increase in the number of oocytes in the gonads when compared to OP50-fed CIA (Figure 3A). This suggests that decreased oocyte production due to caffeine intake was recovered by vitamin B12 supplementation. Oogenesis in *C. elegans* is similar to that in mammals and includes mitosis, transition zone, pachytene, diplotene, and diakinesis from the distal tip region to the proximal region of the gonad. To verify the defects in oocyte development induced by caffeine intake, we observed further changes in the proximal region of the gonads, in which oocytes are located. Both the diplotene and diakinesis stages of the oocytes were counted using DNA staining. The number of diakinesis-stage oocytes was significantly reduced in CIA (Figure 3B–D), which was recovered by vitamin B12 supplementation. This result indicates that vitamin B12 protects oocyte development from caffeine inhibition at the diakinesis stage during oogenesis.

Next, we examined the mitochondria in oocytes. Mitochondrial maturation, the transition from globular to tubular mitochondria, is a critical regulator of germline differentiation during oogenesis in *C. elegans* [33]. We observed transgenic animals that expressed a fusion protein of *C. elegans* mitochondrial import receptor subunit TOMM-20 and GFP as mitochondrial markers in oocytes, the expression of which was induced in germ cells and embryos by the *mex-5* promoter. As shown in Figure 3E, the mitochondrial morphology in the −1 oocyte was altered in CIA compared to that in animals fed a caffeine-free diet. Mitochondria in oocytes from CIA were mostly globular, whereas mitochondria of oocytes from CIA fed the vitamin B12 diet were significantly more tubular in shape (Figure 3E). This morphological change in the mitochondria of oocytes induced by caffeine intake also resulted in the alteration of mitochondrial ROS levels in the oocytes (Figure 3F). Both the DA1877 diet and vitamin B12 supplementation reduced ROS levels, similar to the restoration of mitochondrial shape in oocytes by vitamin B12 in CIA (Figure 3F). These results suggest that caffeine intake inhibits the transition of mitochondrial morphology during oocyte development and causes redox imbalance; however, vitamin B12 has a protective effect on oocyte development in CIA.

Abnormal mitochondrial morphology is associated with the collapse of MMP (ΔΨm). MMP is important for mitochondrial function, including ATP production and mitochondrial dynamics. To further examine whether caffeine intake affects MMP in oocytes, we analyzed the intensity of TMRM fluorescence in CIA oocytes with or without vitamin B12 (Figure 3G). MMP was significantly increased in oocytes with caffeine intake compared to that in animals of caffeine-free diet (Figure 3G). Furthermore, the increased level of TMRM fluorescence was restored by vitamin B12 supplementation, suggesting that vitamin B12 improves MMP in CIA oocytes.

### 3.4. Antioxidants Restore Vitellogenesis and Oocyte Development in Caffeine-Ingested C. elegans, and Mitochondrial Stress Decreases Yolk Protein Production in Oocytes

**Figure 4:**
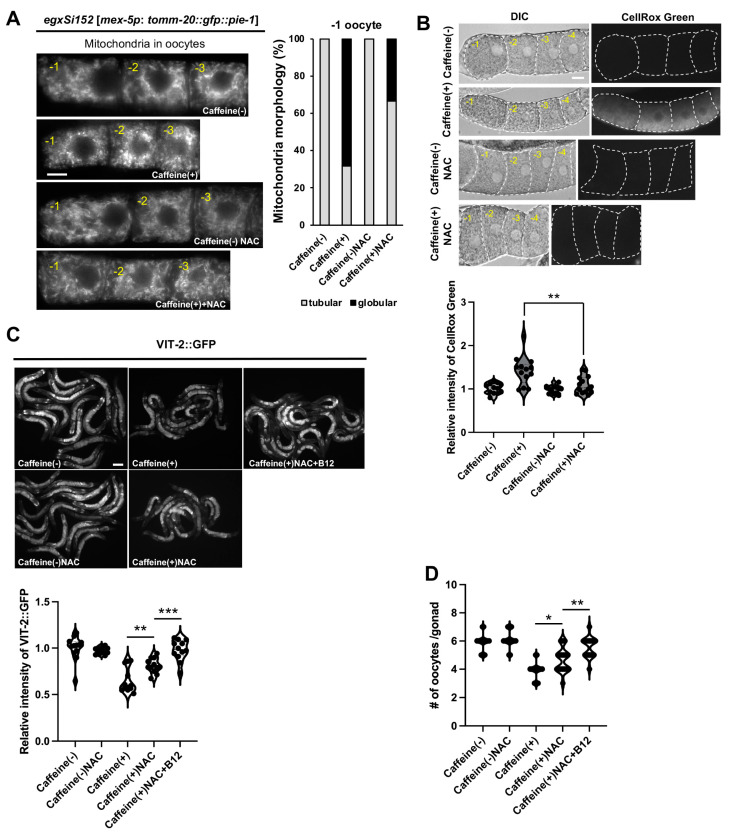
The antioxidant effect of vitamin B12 on oocyte mitochondria and vitellogenin in CIA. (**A**) Comparison of mitochondrial morphology in the oocytes of transgenic animals (EGD623, *egxSi152 [mex-5*p:: *tomm-20*::*gfp*::*pie-1]*) fed with or without caffeine after NAC treatment. Bar, 5 μm. The graph shows the distribution of mitochondrial morphology in the −1 oocyte. (**B**) Representative images and graphs show the relative level of mitochondrial reactive oxygen species using CellROX Green dye in the oocytes of CIA with NAC treatment. Bar, 5 μm. (**C**) Comparison of the relative intensity of VIT-2::GFP in CIA supplemented with NAC or NAC + vitamin B12. Bar, 50 μm. (**D**) The graph shows the number of diakinesis oocytes in CIA supplemented with NAC or NAC + vitamin B12. Data represent mean ± standard deviation. *, *p* < 0.05. **, *p* < 0.01. ***, *p* < 0.001. Number of analyzed animals, *n* ≥ 20 in respective conditions.

Vitamin B12 appears to possess antioxidant properties, and B12 deficiency results in oxidative stress [34]. The increased levels of ROS and TMRM in CIA were reduced by vitamin B12 supplementation, suggesting that vitamin B12 has a function in oxidative stress resistance. To determine whether caffeine-induced mitochondrial alterations and the reduction in vitellogenesis in oocytes were associated with oxidative stress, we observed mitochondrial morphology and ROS levels in CIA oocytes after treatment with the strong antioxidant NAC (Figure 4A). NAC supplementation in CIA partially restored mitochondrial morphology and reduced ROS levels in oocytes (Figure 4A,B). Furthermore, we found that the reduced expression of VIT-2::GFP and oocyte production in CIA increased with NAC supplementation (Figure 4C). These findings indicate that mitochondrial dysfunction, reduced vitellogenesis, and reduced oocyte numbers in CIA were due to oxidative stress. Surprisingly, VIT-2::GFP expression and the number of oocytes increased when vitamin B12 was supplemented with NAC (Figure 4C,D). Collectively, these findings indicate that vitamin B12 exerts antioxidant effects. 

**Figure 5:**
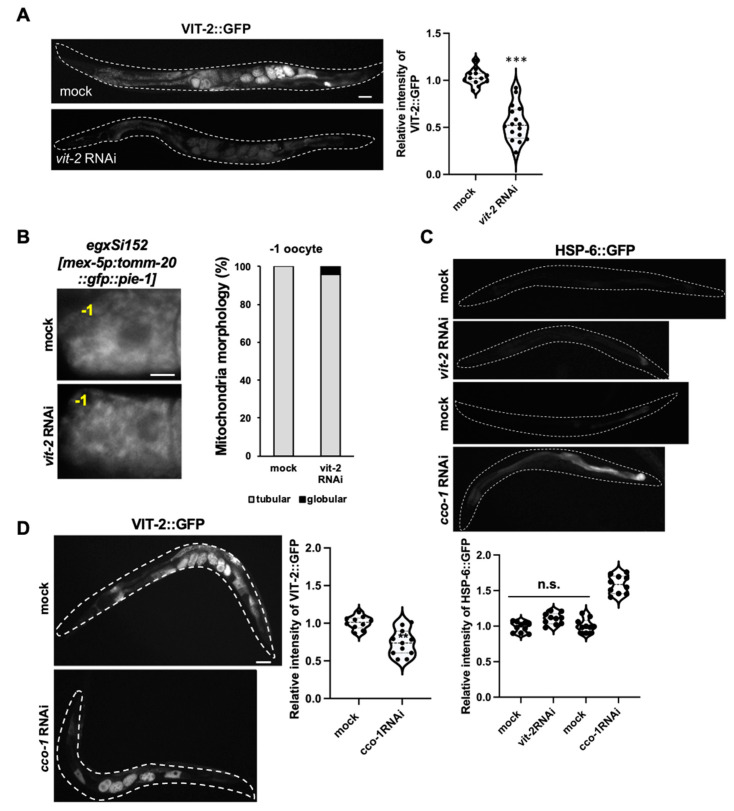
Correlated effects of vitellogenin and mitochondrial stress. (**A**) *vit-2* RNAi effectively suppressed in VIT-2::GFP transgenic animals. Representative images of VIT-2::GFP transgenic animals following *vit-2* RNAi treatment. Bar, 25 μm. ***, *p* < 0.001. Number of analyzed animals, n ≥ 20 in respective conditions. (**B**) The representative images and bar graph show the comparison of mitochondrial morphology in the oocytes of EGD623 (*egxSi152 [mex-5*p:: *tomm-20*::*gfp*::*pie-1]*) transgenic animals treated with mock or *vit-2* RNAi. Bar, 5 μm. (**C**) Representative images and violin plots show the comparison of the relative intensity of HSP-6::GFP treated with mock, *vit-2*, and *cco-1* RNAi. *cco-1* RNAi was used as a positive control to induce the mitochondrial stress response. Bar, 25 μm. (**D**) Representative images and a violin plot show the comparison of the relative intensity of VIT-2::GFP treated with mock and *cco-1* RNAi. Bar, 25 μm. Data represent mean ± standard deviation. n.s., *p* > 0.5. **, *p* < 0.01. Number of analyzed animals, *n* ≥ 20 in respective conditions.

To further investigate the possible correlation between the reduction in vitellogenesis and mitochondrial stress response in CIA, we performed *vit-2* or *cco-1* RNAi on either TOMM-20::GFP or HSP-6::GFP transgenic animals (Figure 5). *cco-1* is a gene that induces the specific mitochondrial stress response, TOMM-20::GFP is a marker of mitochondrial morphology in oocytes, and HSP-6::GFP is used to assess the mitochondrial oxidative stress response in the intestine. After *vit-2* RNAi, the animals showed no significant changes in either oocytes or intestine compared to the control animals (Figure 5A–C), suggesting that the reduction in vitellogenesis did not affect mitochondrial alterations. However, animals treated with *cco-1* RNAi showed reduced levels of VIT-2::GFP expression in contrast to increased HSP-6::GFP expression (Figure 5C,D). These results suggest that a reduction in vitellogenin did not induce mitochondrial alterations, but that vitellogenesis was decreased by mitochondrial stress. 

## 4. Discussion

Nutritional diets are critical regulators of animal growth and development. In particular, the vitamin B group appears to be essential; for example, vitamin B12 deficiency causes defects in development [35]. Here, we found that vitamin B12 supplementation recovered caffeine-induced negative effects on reproduction by reducing mitochondrial oxidative stress and increasing yolk protein levels in oocytes.

**Figure 6:**
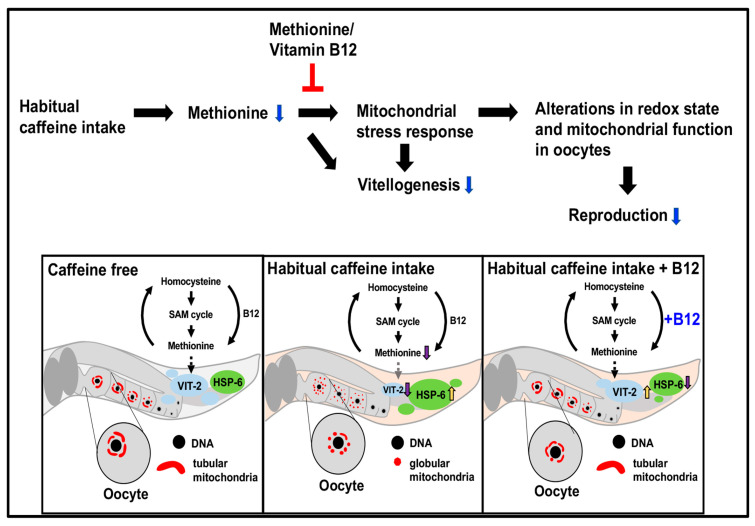
The illustration proposes a working model for this study.

Caffeine intake interfered with morphological changes during mitochondrial maturation in oocytes, whereas vitamin B12 appeared to protect this process (Figure 6). It was recently reported that caffeine can lead to mitochondrial fission through the cAMP/PKA/AMPK signaling pathway, and that caffeine-induced mitochondrial fission is an important process in cell migration in the human endothelial cell system, suggesting that caffeine induces angiogenesis by modulating endothelial mitochondrial dynamics [36]. Consistent with this finding, we found that caffeine intake disrupted the morphological maturation of oocyte mitochondria, resulting in the inhibition of oocyte development. How does caffeine intake affect mitochondrial morphological dynamics and function? Previous studies have shown that caffeine induces the activity of ryanodine receptors—a family of Ca^2+^ release channels in intracellular Ca^2+^ storage/release organelles—and stimulates the release of Ca^2+^ to increase Ca^2+^ concentration in the cytoplasm, forming a complex with calmodulin [37]. An increased level of intracellular Ca^2+^ has been shown to promote mitochondrial fission [38], suggesting that caffeine exerts a negative effect on oocyte development by inhibiting mitochondrial dynamics through the alteration of cytoplasmic Ca^2+^ concentration.

Vitellogenins, which are yolk proteins, are exclusively expressed in the adult intestine and supply nutrients to developing oocytes to support offspring development [39,40]. Recent extensive genetic studies have shown that multiple regulators and pathways are directly or indirectly involved in vitellogenesis in *C. elegans* [40]. Various signaling pathways, such as the insulin/insulin-like growth factor signaling, target of rapamycin, and TGF-β pathways, regulate the expression of vitellogenins in response to environmental conditions [40]. In our previous study, we demonstrated that maternal caffeine intake reduced yolk protein production by regulating *unc-62* expression, which caused the disruption of eggshell integrity and the inhibition of larval development [9]. In this study, we found that the mitochondrial stress response to caffeine intake reduced vitellogenesis in *C. elegans* by markedly decreasing *vit-2* expression following *cco-1* RNAi depletion, but not vice versa. These findings suggest that mitochondria play an important role in the regulation of yolk protein production. Considering the role of the *unc-62* transcription factor [9], there is a possible link between mitochondrial alterations and the nuclear transcriptional regulation of vitellogenesis in CIA. Studies on *Saccharomyces cerevisiae* have revealed a communication network that harmonizes with the functions of the nucleus, cytosol, and mitochondria [41]. Mitochondria-to-nucleus communication is activated by mitochondrial dysfunction, including alterations in MMP, the TCA cycle, and oxidative phosphorylation genes [41]. Mitochondrial defects induce an adaptive transcriptional response that compensates for mitochondrial dysfunction to restore metabolic fitness and cellular homeostasis. Interestingly, hypoxia-inducible factor-1 (HIF-1) mediates adaptive responses to oxidative stress via nuclear translocation and the regulation of gene expression [42]. HIF-dependent regulation in mitochondrial function appears to be dependent on HIF-1 nuclear translocation or by directly targeting mitochondria [43,44]. Therefore, one intriguing hypothesis is that mitochondrial stress in oocytes caused by caffeine intake is conveyed to the nucleus to repress the expression of *unc-62*, resulting in a decrease in vitellogenesis, and thus a reduction in reproduction, which remains to be determined. 

*C. elegans* has emerged as an important model in the field of environmental toxicology. Although we found that caffeine intake showed the alterations in mitochondrial function during oocyte development in *C. elegans*, there are limitations in applying it to directly the physiological correlation between *C. elegans* and humans. Moreover, because of the drug delivery methods for *C. elegans*, which were indirect and often varied from one to another [45,46,47,48], a poor absorption issue through the cuticle, and perhaps changes in behavior that impede the uptake of many chemicals, the caffeine concentration treated to *C. elegans* in this study may be variable. Therefore, to clarify the physiological correlation with human by caffeine intake, it is required to measure the actual absorption of caffeine intake in *C. elegans*. 

In this study, we demonstrated that morphological and functional changes in mitochondria caused by caffeine intake affected oocyte development, which was recovered by vitamin B12 supplementation. These findings suggest that vitamin B12 plays a protective role in mitochondrial function and morphological maturation during oocyte development. Furthermore, we demonstrated the beneficial effects of vitamin B12 in CIA on vitellogenesis at the adult stage and the early developmental growth of *C. elegans*, indicating that vitamin B12 is required at all developmental stages. Taken together, this study provides important insights into the beneficial role of vitamin B12 supplementation in mitochondrial function under stressful environments due to various dietary conditions, and thus improves oocyte development through yolk protein production.

## 5. Conclusions

In summary, we found beneficial effects of vitamin B12 in vitellogenesis and the mitochondrial stress response during oogenesis in CIA. Moreover, both the decreased number of developing oocytes and the elevated levels of ROS in oocytes from CIA were recovered by vitamin B12 supplementation through the improved mitochondrial function. This study shows that vitamin B12 supplementation plays a protective role in vitellogenesis and oocyte development by the maintenance of mitochondrial function in CIA.

## Data Availability

Data will be made freely available upon request to the corresponding author Y.-H. Shim.

## References

[1] Panagiotou M., Meijer M., Meijer J.H., Deboer T. (2019). Effects of Chronic Caffeine Consumption on Sleep and the Sleep Electroencephalogram in Mice. J. Psychopharmacol..

[2] Haraguchi A., Yamazaki T., Ryan C., Ito K., Sato S., Tamura K., Sekiguchi M., Cao S., Shibata S. (2022). Caffeine Suppresses High-Fat Diet-Induced Body Weight Gain in Mice Depending on Feeding Timing. J. Funct. Foods.

[3] Li H., Roxo M., Cheng X., Zhang S., Cheng H., Wink M. (2019). Pro-Oxidant and Lifespan Extension Effects of Caffeine and Related Methylxanthines in *Caenorhabditis elegans*. Food Chem. X.

[4] Leung M.C.K., Williams P.L., Benedetto A., Au C., Helmcke K.J., Aschner M., Meyer J.N. (2008). *Caenorhabditis elegans*: An Emerging Model in Biomedical and Environmental Toxicology. Toxicol. Sci..

[5] Min H., Kawasaki I., Gong J., Shim Y.H. (2015). Caffeine Induces High Expression of Cyp-35A Family Genes and Inhibits the Early Larval Development in *Caenorhabditis elegans*. Mol. Cells.

[6] Al-Amin M., Kawasaki I., Gong J., Shim Y.H. (2016). Caffeine Induces the Stress Response and Up-Regulates Heat Shock Proteins in *Caenorhabditis elegans*. Mol. Cells.

[7] Min H., Youn E., Kawasaki I., Shim Y.H. (2017). Caffeine-Induced Food-Avoidance Behavior Is Mediated by Neuroendocrine Signals in *Caenorhabditis elegans*. BMB Rep..

[8] Min H., Youn E., Shim Y.H. (2021). Long-Term Caffeine Intake Exerts Protective Effects on Intestinal Aging by Regulating Vitellogenesis and Mitochondrial Function in an Aged *Caenorhabditis elegans* Model. Nutrients.

[9] Min H., Youn E., Shim Y.H. (2020). Maternal Caffeine Intake Disrupts Eggshell Integrity and Retards Larval Development by Reducing Yolk Production in a *Caenorhabditis elegans* Model. Nutrients.

[10] Greenstein D. (2005). Control of Oocyte Meiotic Maturation and Fertilization. WormBook.

[11] Kimble J., Sharrock W.J. (1983). Tissue-Specific Synthesis of Yolk Proteins in Caenorhabditis elegans. Dev. Biol..

[12] Sieber M.H., Thomsen M.B., Spradling A.C. (2016). Electron Transport Chain Remodeling by GSK3 during Oogenesis Connects Nutrient State to Reproduction. Cell.

[13] Ben-Shlomo I., Younis J.S. (2014). Basic Research in PCOS: Are We Reaching New Frontiers?. Reprod. Biomed. Online.

[14] Mayer S.B., Evans W.S., Nestler J.E. (2015). Polycystic Ovary Syndrome and Insulin: Our Understanding in the Past, Present and Future. Women’s Health.

[15] Martínez-Reyes I., Chandel N.S. (2020). Mitochondrial TCA Cycle Metabolites Control Physiology and Disease. Nat. Commun..

[16] Li Q., Hoppe T. (2023). Role of Amino Acid Metabolism in Mitochondrial Homeostasis. Front. Cell Dev. Biol..

[17] Catanesi M., Brandolini L., D’Angelo M., Benedetti E., Tupone M.G., Alfonsetti M., Cabri E., Iaconis D., Fratelli M., Cimini A. (2021). L-Methionine Protects against Oxidative Stress and Mitochondrial Dysfunction in an in Vitro Model of Parkinson’s Disease. Antioxidants.

[18] Shafer D.J., Carey J.B., Prochaska J.F. (1996). Effect of Dietary Methionine Intake on Egg Component Yield and Composition. Poult. Sci..

[19] Patton R.A. (2010). Effect of Rumen-Protected Methionine on Feed Intake, Milk Production, True Milk Protein Concentration, and True Milk Protein Yield, and the Factors That Influence These Effects: A Meta-Analysis. J. Dairy Sci..

[20] Zhou G., Huang C., Xing L., Li L., Jiang Y., Wei Y. (2020). Methionine Increases Yolk Production to Offset the Negative Effect of Caloric Restriction on Reproduction without Affecting Longevity in *C. elegans*. Aging.

[21] Olteanu H., Banerjee R. (2001). Human Methionine Synthase Reductase, a Soluble P-450 Reductase-like Dual Flavoprotein, Is Sufficient for NADPH-Dependent Methionine Synthase Activation. J. Biol. Chem..

[22] Froese D.S., Fowler B., Baumgartner M.R. (2019). Vitamin B12, Folate, and the Methionine Remethylation Cycle—Biochemistry, Pathways, and Regulation. J. Inherit. Metab. Dis..

[23] Shiraki N., Shiraki Y., Tsuyama T., Obata F., Miura M., Nagae G., Aburatani H., Kume K., Endo F., Kume S. (2014). Methionine Metabolism Regulates Maintenance and Differentiation of Human Pluripotent Stem Cells. Cell Metab..

[24] Dai Z., Mentch S.J., Gao X., Nichenametla S.N., Locasale J.W. (2018). Methionine Metabolism Influences Genomic Architecture and Gene Expression through H3K4me3 Peak Width. Nat. Commun..

[25] Brenner S. (1974). The Genetics of *Caenorhabditis elegans*. Genetics.

[26] Grant B., Hirsh D. (1999). Receptor-mediated endocytosis in the *Caenorhabditis elegans* oocyte. Mol. Biol. Cell.

[27] Yoneda T., Benedetti C., Urano F., Clark S.G., Harding H.P., Ron D. (2004). Compartment-specific perturbation of protein handling activates genes encoding mitochondrial chaperones. J. Cell Sci..

[28] Fan X., De Henau S., Feinstein J., Miller S.I., Han B., Frøkjær-Jensen C., Griffin E.E. (2020). SapTrap Assembly of *Caenorhabditis elegans* MosSCI Transgene Vectors. G3.

[29] Min H., Lee M., Cho K.S., Lim H.J., Shim Y.-H. (2021). Nicotinamide Supplementation Improves Oocyte Quality and Offspring Development by Modulating Mitochondrial Function in an Aged *Caenorhabditis elegans* Model. Antioxidants.

[30] Tabara H., Grishok A., Mello C.C. (1998). RNAi in *C. elegans*: Soaking in the genome sequence. Science.

[31] Kamath R.S., Martinez-Campos M., Zipperlen P., Fraser A.G., Ahringer J. (2001). Effectiveness of Specific RNA-Mediated Interference through Ingested Double-Stranded RNA in *Caenorhabditis elegans*. Genome Biol..

[32] Brooks K.K., Liang B., Watts J.L. (2009). The influence of bacterial diet on fat storage in *C. elegans*. PLoS ONE.

[33] Charmpilas N., Tavernarakis N. (2020). Mitochondrial Maturation Drives Germline Stem Cell Differentiation in *Caenorhabditis elegans*. Cell Death Differ..

[34] van de Lagemaat E.E., de Groot L.C.P.G.M., van den Heuvel E.G.H.M. (2019). Vitamin B 12 in Relation to Oxidative Stress: A Systematic Review. Nutrients.

[35] Casella E.B., Valente M., De Navarro J.M., Kok F. (2005). Vitamin B12 Deficiency in Infancy as a Cause of Developmental Regression. Brain Dev..

[36] Wang L.-T., He P.-C., Li A.-Q., Cao K.-X., Yan J.-W., Guo S., Jiang L., Yao L., Dai X.-Y., Feng D. (2021). Caffeine Promotes Angiogenesis through Modulating Endothelial Mitochondrial Dynamics. Acta Pharmacol. Sin..

[37] Echeverri D., Montes F.R., Cabrera M., Galán A., Prieto A. (2010). Caffeine’s Vascular Mechanisms of Action. Int. J. Vasc. Med..

[38] Hom J., Yu T., Yoon Y., Porter G., Sheu S.S. (2010). Regulation of Mitochondrial Fission by Intracellular Ca^2+^ in Rat Ventricular Myocytes. Biochim. Biophys. Acta Bioenerg..

[39] Romano M., Rosanova P., Anteo C., Limatola E. (2004). Vertebrate Yolk Proteins: A Review. Mol. Reprod. Dev..

[40] Perez M.F., Lehner B. (2019). Vitellogenins—Yolk Gene Function and Regulation in *Caenorhabditis elegans*. Front. Physiol..

[41] Guaragnella N., Coyne L.P., Chen X.J., Giannattasio S. (2018). Mitochondria–Cytosol–Nucleus Crosstalk: Learning from *Saccharomyces cerevisiae*. FEMS Yeast Res..

[42] Schofield C.J., Ratcliffe P.J. (2005). Signalling Hypoxia by HIF Hydroxylases. Biochem. Biophys. Res. Commun..

[43] Semenza G.L. (2012). Hypoxia-Inducible Factors in Physiology and Medicine. Cell.

[44] Li H.S., Zhou Y.N., Li L., Li S.F., Long D., Chen X.L., Zhang J.B., Feng L., Li Y.P. (2019). HIF-1α Protects against Oxidative Stress by Directly Targeting Mitochondria. Redox Biol..

[45] Petrascheck M., Ye X., Buck L.B. (2007). An antidepressant that extends lifespan in adult *Caenorhabditis elegans*. Nature.

[46] Melov S., Ravenscroft J., Malik S., Gill M.S., Walker D.W., Clayton P.E., Wallace D.C., Malfroy B., Doctrow S.R., Lithgow G.J. (2000). Extension of life-span with superoxide dismutase/catalase mimetics. Science.

[47] Zarse K., Ristow M. (2008). Antidepressants of the serotonin-antagonist type increase body fat and decrease lifespan of adult *Caenorhabditis elegans*. PLoS ONE.

[48] Keaney M., Gems D. (2003). No increase in lifespan in *Caenorhabditis elegans* upon treatment with the superoxide dismutase mimetic EUK-8. Free Radic. Biol. Med..

